# Ceramide from sphingomyelin hydrolysis induces neuronal differentiation, whereas de novo ceramide synthesis and sphingomyelin hydrolysis initiate apoptosis after NGF withdrawal in PC12 Cells

**DOI:** 10.1186/s12964-021-00767-2

**Published:** 2022-01-31

**Authors:** Terri Kagan, Gloria Stoyanova, Richard A. Lockshin, Zahra Zakeri

**Affiliations:** 1 Department of Biology, Queens College of the City University of New York, Flushing, NY USA; 2 St. Johns University, Jamaica, NY USA

**Keywords:** Cell death, Apoptosis, Sphingomyelin, Ceramide, Nerve growth factor

## Abstract

**Background:**

Ceramide, important for both neuronal differentiation and dedifferentiation, resides in several membranes, is synthesized in the endoplasmic reticulum, mitochondrial, and nuclear membranes, and can be further processed into glycosphingolipids or sphingomyelin. Ceramide may also be generated by hydrolysis of sphingomyelin by neutral or acidic sphingomyelinases in lysosomes and other membranes. Here we asked whether the differing functions of ceramide derived from different origins.

**Methods:**

We added NGF to PC12 cells and to TrkA cells. These latter overexpress NGF receptors and are partially activated to differentiate, whereas NGF is required for PC12 cells to differentiate. We differentiated synthesis from hydrolysis by the use of appropriate inhibitors. Ceramide and sphingomyelin were measured by radiolabeling.

**Results:**

When NGF is added, the kinetics and amounts of ceramide and sphingomyelin indicate that the ceramide comes primarily from hydrolysis but, when hydrolysis is inhibited, can also come from neosynthesis. When NGF is removed, the ceramide comes from both neosynthesis and hydrolysis.

**Conclusion:**

We conclude that the function of ceramide depends heavily on its intracellular location, and that further understanding of its function will depend on resolving its location during changes of cell status.

**Graphical Abstract:**

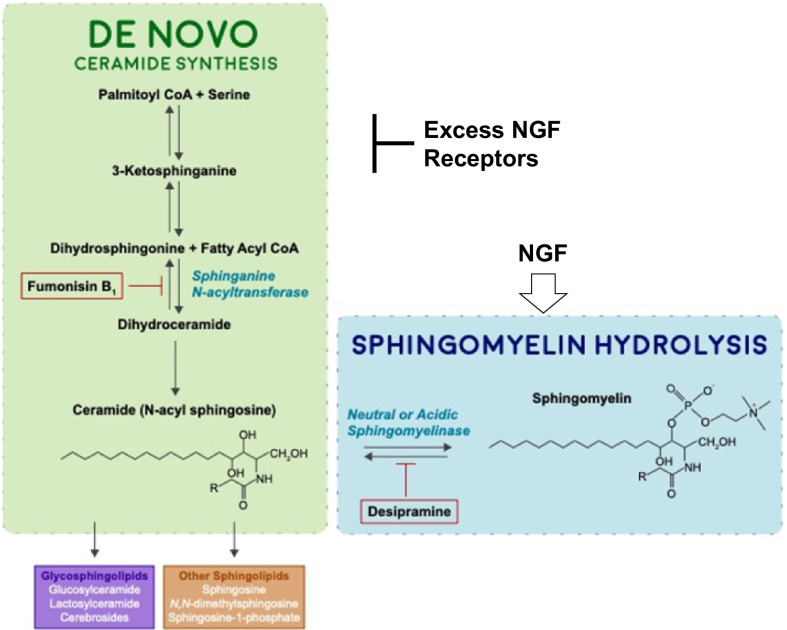

**Video Abstract**

**Supplementary Information:**

The online version contains supplementary material available at 10.1186/s12964-021-00767-2.

## Plain English summary

Ceramide and sphingomyelin reportedly are important both for differentiation of nerve cells and for their death. We studied PC12 cells, which can differentiate into neuron-like cells in the presence of nerve growth factor and cells that overexpress receptors for nerve growth factor. By combining various inhibitors, we conclude that in the presence of nerve growth factor ceramide comes from hydrolysis of sphingomyelin, but when nerve growth factor is removed and the cells atrophy and die, sphingomyelin comes from both neosynthesis and hydrolysis.

**Supplementary Information:**

The online version contains supplementary material available at 10.1186/s12964-021-00767-2.

## Background

Neuron differentiation, proliferation, and survival in the central and peripheral nervous systems depend on the interactions between receptor/neurotrophin pairs such as the interactions of nerve growth factor (NGF) with its receptors p75 (p75^NTR^) and the tropomyosin receptor kinase A (TrkA) [1, 2]. The question we ask is how are these receptors involved in the differentiation of PC12 cells into neuronal types, and how do they function when the cells are deprived of nerve growth factor (NGF)?

These receptors function differently, with consequences for the biology of cells such as PC12 cells. The pan receptor p75^NTR^ has a lower affinity for NGF and is compatible with multiple neurotrophins. TrkA has higher affinity for NGF and can carry NGF into the cell [3]. The binding of NGF homodimer to TrkA stimulates TrkA dimerization and phosphorylation of tyrosine residues on the intracellular domain, providing docking sites for adaptor proteins to relay a signal [4]. The signaling activity of TrkA is complex and has been well studied. Pathways essential to proliferation and differentiation as mediated by NGF thru TrkA include the Ras pathway, mitogen-activated protein kinase (MAPK) pathway, phospholipase C (PLC-γ), and the phosphatidylinositol 3-kinase (PI3K) pathway [4]. PI3K phosphorylates the downstream target Akt/protein kinase B (PKB) and activation of the transcription factor NF-kB [5]. Unlike TrkA, p75^NTR^ does not have autocatalytic kinase activity and is believed to rely on the intracellular kinase activity of TrkA for signal transduction [6].

Activation of NF-kB and neuroprotection by NGF is dependent on p75^NTR^ expression in PC12 cells [5], indicating the importance of this receptor for these functions. p75^NTR^ activation also turns on production of the sphingolipid ceramide, which functions as a second messenger [7].

Ceramides have three pathways to synthesis; de novo on the endoplasmic reticulum with palmitoyl-CoA and serine as the starting materials; from sphingomyelin (SM) hydrolysis; or from the endosomal salvage pathway [8]. Once ceramide is synthesized, it can either be retained to yield cellular sphingomyelin in the plasma membrane or the lysosomal membranes. Interconversion between sphingomyelin and ceramides is possible [8]. Ceramide can be produced by hydrolysis from acidic sphingomyelinases (aSMases) or neutral sphingomyelinases (nSMases) [9].

Increase in ceramide promotes differentiation and growth in various cell types [10, 11]. However, ceramide second messengers can exert an antagonistic effect, causing apoptosis likely through increased phosphorylation of c-Jun N-terminal Kinase (JNK) [12]. NGF binding to TrkA and downstream phosphorylation of Akt (PKB) promote survival and inhibit apoptosis [13]. In contrast, at least exogenous ceramide can induce apoptosis, operating at all levels of the mitochondrial signaling cascade [14, 15]. Ceramide deriving from p75^NTR^ activation and downstream ceramide synthesis can also activate apoptosis, independently of TrkA [16]. Ceramides produced by neutral SMases are required for cell death in oligodendrocytes and hippocampal neurons [17, 18], acting through JNK [19] and perhaps the ceramide-binding pocket of voltage-dependent anion channel 2 (VDAC2) [20, 21].

The integration of TrkA and ceramide signaling can be studied in PC12 cells, which differentiate into neurons following NGF stimulation. PC12 cells have both high-affinity TrkA and low-affinity p75^NTR^ binding sites for NGF [22]. Increases in sphingomyelin hydrolysis are associated with differentiation following the activation of p75^NTR^, while the TrkA receptor has been reported to have an antagonistic effect [10, 23]. PC12 cells that overexpress the TrkA receptor produce neuronal characteristics faster than wild-type [24], suggesting that the ceramide generated from sphingomyelin hydrolysis is not essential to neuronal differentiation. Because of the complicated and sometimes contradictory observations, we elected to explore the extent to which ceramide derived from neosynthesis and from hydrolysis determined the differentiation or death of PC12 and TrkA cells. The Trk 6–24 PC12 cell line (TrkA) that we used overexpresses the constitutive TrkA receptor 20-fold. PC12 cells overexpressing TrkA show a basal, constitutive, activation of kinase but grow more slowly than PC12 cells [25, 26], are resistant to NGF-withdrawal-induced apoptosis [24]. Our results suggest that differentiation is supported primarily by ceramide derived from hydrolysis of sphingomyelin, whereas when NGF is withdrawn and cells atrophy and die, ceramide comes from both hydrolysis and from neosynthesis.

## Methods

### Cell culture and treatment

Rat adrenal pheochromocytoma (PC12) cells were obtained from the American Type Culture Collection (ATCC). Trk 6–24 PC12 cells (TrkA) were a gift of Dr. Raymond Birge (UMDNJ). RPMI, DMEM (Dulbecco’s Modified Eagle Medium), penicillin, streptomycin, G418 (gentamycin), fetal bovine serum (FBS), and horse serum were obtained from Gibco BRL. Nerve growth factor (NGF) was from Harlan Bioproducts. PC12 cells were maintained in RPMI-1640 (Roswell Park Memorial Institute formulation) supplemented with 5% FBS, 10% horse serum, 50 U/ml penicillin, and 100 μg/ml streptomycin and TrkA cells were maintained in DMEM with 2% FBS, 1% horse serum, 50 U/ml penicillin, 100 μg/ml streptomycin and 100 μg/ml G418 in a humidified atmosphere of 95% air and 5% CO_2_ at 37°C. Naïve cells were induced to differentiate and maintained by transfer to DMEM supplemented with 2% FBS, 1% horse serum, 0.05 ng/ml NGF, 50 U/ml penicillin and 100 μg/ml streptomycin. At the end of the incubation cells were pelleted, washed once with PBS (pH 7.4), and examined.

For NGF deprivation studies, cells were washed once in phosphate buffered saline (PBS, 137 mM NaCl, 2.7 mM KCl, 4.3 mM Na_2_HPO_4_·7H_2_O, 1.4 mM KH_2_PO_4_, pH 7.4), and replaced in 100% DMEM containing antibiotics for 1, 3, 6, 12, 18, or 24 h. For inhibitor studies, fumonisin B_1_ (FB_1_) or desipramine (Sigma-Aldrich; St. Louis, MO) were diluted in 0.9% NaCl and added to the cultures commencing the first day of treatment. Control cells received 0.9% NaCl alone. Final solvent concentrations did not exceed 0.1% or have detectable effects. Specific concentrations were established from dose–response curves (data not shown) and for the results reported here FB_1_ was used at 50 μM and desipramine at 7.5–15 μM.

### Quantification of apoptotic cells

To assess the viability of treated cells, we directly counted cells with a hemocytometer after exposure to trypan blue [26]. The percentage of cells excluding the dye was calculated with respect to the corresponding control and confirmed by 3–4 independent trials. Viability was further confirmed by commercial LIVE/DEAD® assay kit (Molecular Probes; Eugene, OR). To detect morphological changes in the nuclei of treated cells we used the DNA fluorochrome *bis*-benzimide (Hoechst 33,258-Sigma-Aldrich; St. Louis, MO) as described [27]. Only nuclei with supercondensed chromatin at the nuclear periphery or nuclei fragmented into smaller dense bodies were considered apoptotic. In situ detection of actual DNA fragmentation in cells grown on coverslips or slides was also performed using the ApopTag® kit (Intergen; Purchase, NY) essentially as described [28].

### Lipid studies

The intracellular level of ceramide by the *E. coli* DG kinase assay was determined as described [38].

The intracellular level of ceramide was determined by the *E. coli* DG kinase assay using the Bligh-Dyer method essentially as described [29]. Cells were collected by scraping and washed twice with PBS (pH 7.4). Each tube received approximately 3–4 × 10^6^ cells, and each point was repeated twice. The cells were lysed in 1 ml chloroform/methanol/1 N HCl (Kill solution; 100/100/1, v/v/v). The organic phase was separated by adding 270 μl of buffered saline solution (BSS, 135 mM NaCl, 4.5 mM KCl, 1.5 mM CaCl_2_, 0.5 mM MgCl_2_, 5.6 mM glucose and 10 mM HEPES, pH 7.2) and 30 μl of 100 mM EDTA and vortexing. The phases were separated by 5 min of centrifuging and then the lower organic phase (total cellular lipids) was removed to a clean tube and dried under a stream of N_2_ set at 20–25 psi for 10–15 min in a hot water bath.

To eliminate glycerophospholipids, the extracted lipid film was subjected to mild alkaline hydrolysis (0.1 M methanolic potassium hydroxide) for 1 h at 37 °C in a water bath. The organic phase was re-isolated by adding 270 μl of BSS (pH 7.2), 30 μl of 100 mM EDTA and 500 μl CHCl_3_ followed by vortexing. The phases were again separated as above.

Cellular lipids were labeled with reaction mixture (Cardiolipin (10 mg/ml from Avanti Polar Lipids), DETAPAC (1 mM from Sigma-Aldrich, St. Louis, MO), N-Octyl-β-D-glucopyranoside (825 mM from Calbiochem), 2X reaction buffer (NaCl 100 mM, imidazole 100 mM, EDTA 2 mM, MgCl_2_ 25 mM; pH 6.5), imidazole/DETAPAC (10 mM/1 mM), ATP (100 mM), Escherichia coli diacylglycerol (DG) kinase (1 mg/ml from Calbiochem), [γ-32P] dATP (111 Bq/μmol from Amersham Corp.) for 30 min at room temperature. The reaction was stopped and ceramide-1-phosphate was extracted by adding 1 ml Kill solution and 170 μL BSS, 30 μL 100 mM EDTA to each tube. Tubes were vortexed and spun in a centrifuge for 5 min and the lower organic phase was again transferred to clean tubes and dried under N_2_. The lipid film was finally resuspended in 50 μL CHCl_3_:MeOH (1:1). 30 μL ceramide was loaded into each lane of a TLC plate (6 nm silica gel plates of 0.25 thickness from Whatman) along with known concentrations of ceramide (natural ceramide type III from bovine brain from Sigma-Aldrich, St. Louis, MO) as described [29]. The plates were chromatographed in a tank with CHCl_3_:MeOH:HAc (65:15:5) for 45–75 min until the solvent front was 1–2 cm from the top. The plate was then dried and exposed to X ray film for 24 h. Finally, the radioactive ceramide bands were excised and counted by scintillation, or scanned with STORM phosphoimager and quantified using IMAGEQUANT software. Counts per minute were converted to pmols by reading values off a simultaneously run standard curve consisting of known quantities of ceramide.

The remaining lipid solution was dried under N_2_ and used to normalize ceramide levels to cellular phosphate as described [30]. NaH_2_PO_4_ standards were prepared (0–80 nM) in CHCl_3_:MeOH (1:1). 600 μl ashing buffer (9:1:40 10 N H_2_SO_4_:70% HClO_4_:H_2_O) is added to each tube and tubes are left at 160 °C overnight or 220ºC for 2 h. 900 μL H_2_O, 500 μL ammonium molybdate (0.9% w/v) and 200 μL fresh ascorbic acid (9.0% w/v) were added and tubes were vortexed, left at 45ºC for 30 min and read in a spectrophotometer at 820 mM. All results displayed are the average of at least three independent sets of experiments.

Natural ceramide (type III from bovine brain), from Sigma-Aldrich (St. Louis, MO) was used as a control. N-octanoyl-D-erythro-sphingosine (D-e-C_8_-cer) was purchased from Biomol. Cardiolipin (beef heart) was from Avanti Polar Lipids. Radioactivity was quantified as above. Ceramide levels were further normalized to cellular phosphate as described [30]. Total cellular sphingomyelin was also determined and normalized essentially as described [30] using [^3^H] choline chloride from American Radiolabeled Chemicals. Ceramide was calculated as picomoles per μg PO_4_, whereas sphingomyelin was reported relative to control.

### Sphingomyelin mass assay

Total cellular sphingomyelin (SM) was determined essentially as described by [31]. SM was labeled with 50 μCi (1.85 × 10^6^ Bq) [3H] choline chloride (American Radiolabeled Chemicals) for 48 h before treatment. After treatment, media was removed and cells (5–6 × 10^5^) were collected by scraping in 3 ml MeOH:CHCl_3_ (2:1).

Lipids were extracted using the Bligh-Dyer method essentially as described above. 800 ml H_2_O was added to create a monophase, and tubes were spun for 5 min at 2000 g to remove large debris. The supernatant was transferred to a clean tube, CHCl_3_:H_2_O (1:1) added, and tubes vortexed. After spinning for 5 min at 2000 g, the upper inorganic phase was partially removed and lower organic phase was transferred to a new tube and dried under N_2_ (20–25 psi for 10–20 min). The lipid film was subjected to mild alkaline hydrolysis by addition of 250 μl methanolic NaOH (2 N) for 2 h at 37 °C in a water bath. The base solution was neutralized by the addition of 250 μL HCl (2 N) and the lipids re-extracted in 430 μL H_2_O, 500 μL CHCl_3_:MeOH (2:1) and 850 μL CHCl_3_ followed by vortexing and centrifuging for 5 min. The lower organic phase was again collected into a fresh tube and dried under N_2_ as above. The lipid film was resuspended in 70 μl CHCl_3_, of which 50 μl was spotted onto TLC plates.

Plates were developed in a tank with CHCl_3_:MeOH:HAc:H_2_O (50:30:8:5) and run until solvent front was 1–2 cm from top. The plate was then dried and exposed to iodine vapor and the radioactive sphingolipid bands excised and counted by scintillation. Because radioactive sphingomyelin standards are not employed by this assay, results cannot be directly quantified to pmols/cells and are therefore represented as percent of control values. All results are the average of at least three independent sets of experiments and have been further normalized to total cellular phosphate as described above using the lipid solution remaining after the TLC plates were loaded.

### Statistics

The means of duplicates of at least three separate experiments, or duplicate counts of at least 250 cells for each of three separate experiments, were analyzed by Student’s two-sample t-test. Significance was accepted at *p* < 0.05 (one asterisk in figures) and *p* < 0.01 (two asterisks in figures).

## Results

**Fig. 1:**
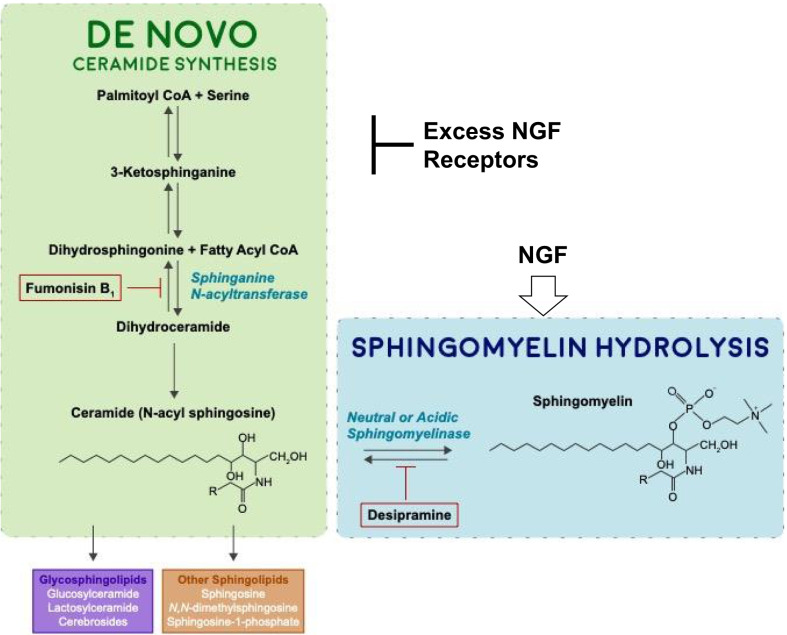
The biosynthesis and metabolism of ceramide. Ceramide is both an end product and penultimate precursor for various sphingolipids, such as sphingomyelin, which is synthesized in the plasma membrane and glycosphingolipids. The enzymes involved in ceramide metabolism are shown in italics, compounds found or synthesized in the endoplasmic reticulum are in boldface type those found or synthesized in the Golgi apparatus are in normal type. The inhibitors used in our studies are outlined in red. (Adapted from [50]). Hypothesized locations of actions of excess NGF and of extra TRK receptors in the TrkA cells, addressed in Discussion, are also indicated

The general scheme of the pathways for production and consumption of ceramide is shown in Fig. 1. Both the synthetic and hydrolytic pathways are reversible, but the inhibitors are considered to block both, suppressing the interconversion, with Fumonisin B_1_ blocking neosynthesis and Desipramine hydrolysis of sphingomyelin. The hypothesized roles of NGF (Nerve Growth Factor) and excess NGF receptors, presented as data in the paper, are also shown.

### Control of ceramide concentration: addition of NGF

**Fig. 2:**
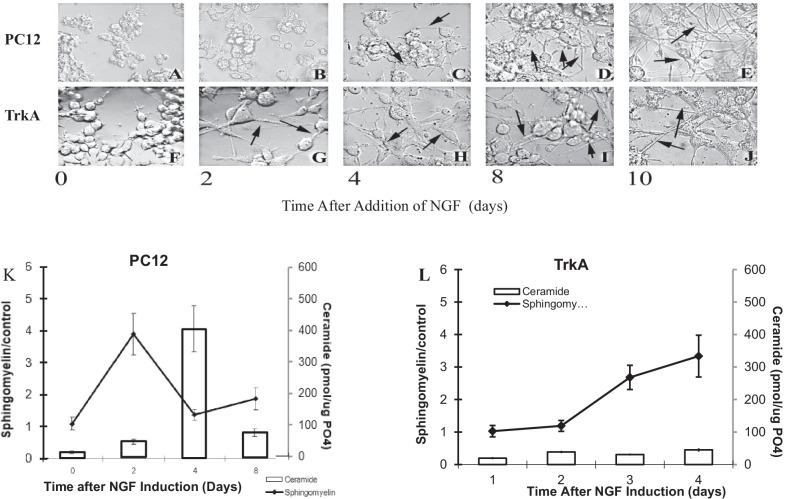
The effect of NGF on differentiation in PC12 and TrkA cells. PC12 (**A**–**E**) and TrkA (**F**–**J**) cells were induced to differentiate with 0.05 ng/ml NGF. Neuronal morphology (shrunken soma and long extended neurites that synapse on other cells) is indicated with arrows (×400 magnification). PC12 (**K**) and TrkA (**L**) cells were treated as above, lipids were extracted and ceramide and SM levels quantified by the DG kinase assay and SM mass assay as described in Materials and Methods. Each value represents the mean of duplicate determinations from at least three experiments; bars, SEM

In the presence of NGF, PC12 cells begin to show neurites within 4 days and continue to differentiate into neuron-like cells for the next week (Fig. 2a–e). During the early phases of this differentiation, they first (within 2 days) transiently quadruple their relative sphingomyelin content, followed by a 20–50-fold, likewise transient, increase in ceramide (Fig. 2k). TrkA cells, which can spontaneously differentiate and therefore show neurites within two days of passage (Fig. 2f–j), more gradually accumulate sphingomyelin and do not show a burst in ceramide concentration (Fig. 2l).

**Fig. 3:**
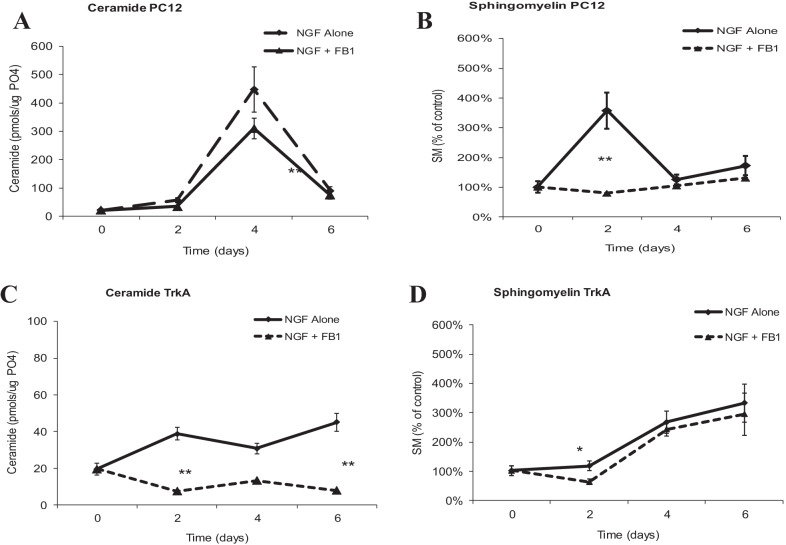
**A** Time course for ceramide generation in PC12 cells treated as above. Lipids were extracted and ceramide levels quantified by the DG kinase assay as described in Materials and Methods. **B** Time course for SM generation in PC12 cells treated as above. Lipids were extracted and SM levels quantified by the SM mass assay as described in Materials and Methods. **C** Time course for ceramide generation in TrkA cells treated as above. Lipids were extracted and ceramide levels quantified by the DG kinase assay as described in Materials and Methods. **D** Time course for SM generation in TrkA cells treated as above. Lipids were extracted and SM levels quantified by the SM mass assay as described in Materials and Methods. Each value represents the mean of duplicate determinations from at least three experiments; bars, SEM; **p ≤ 0.01

**Fig. 4:**
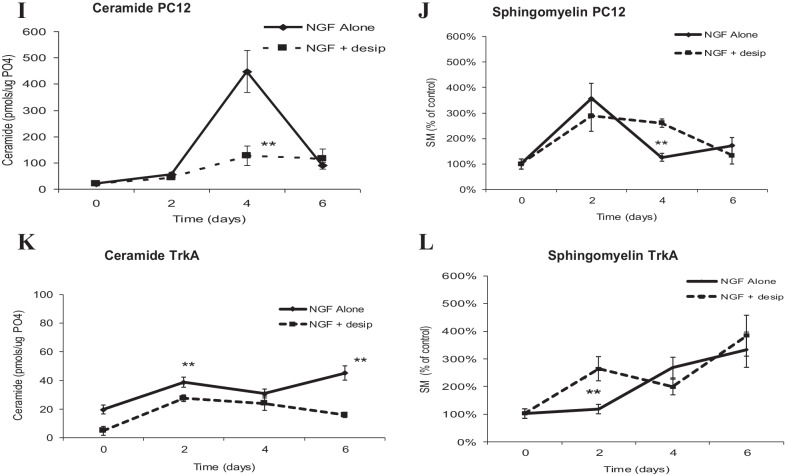
The effect of desipramine on differentiation of PC12 and TrkA cells. **A** Time course for ceramide generation in PC12 cells treated as above. Lipids were extracted and ceramide levels quantified by the DG kinase assay as described in Materials and Methods. **B** Time course for SM generation in PC12 cells treated as above. Lipids were extracted and SM levels quantified by the SM mass assay as described in Materials and Methods. **C** Time course for ceramide generation in TrkA cells treated as above. Lipids were extracted and ceramide levels quantified by the DG kinase assay as described in Materials and Methods. **D** Time course for SM generation in TrkA cells treated as above. Lipids were extracted and SM levels quantified by the SM mass assay as described in Materials and Methods. Each value represents the mean of duplicate determinations from at least three experiments; bars, SEM; **p ≤ 0.01

Blocking of neosynthesis fails to prevent differentiation of either PC12 cells or TrkA cells In PC12 cells, ceramide concentration is little affected, while, surprisingly, sphingomyelin fails to accumulate (Fig. 3a, b), suggesting that in the presence of NGF, sphingomyelin has a relatively high turnover rate, being converted to molecules other than ceramide, and that its pool is replenished by neosynthesis of ceramide. In TrkA cells, blocking of neosynthesis causes ceramide to drop, while sphingomyelin is relatively unaffected (Fig. 3c, d), suggesting that in these chronically stimulated cells ceramide is provided by neosynthesis while sphingomyelin is maintained through other mechanisms. Blocking hydrolysis of sphingomyelin by desipramine, primarily by inhibiting neutral sphingomyelinases, suppresses neuronal differentiation in both PC12 cells and in TrkA cells. In this situation, the ceramide peak in PC12 cells is blocked, while there is little overall impact on the ceramide in TrkA cells or on sphingomyelin in either type of cell (Fig. 4a–d). This result also suggests that NGF stimulates ceramide produced by neosynthesis, while TrkA cells are relatively unaffected by NGF.

### Control of ceramide concentration: removal of NGF

**Fig. 5:**
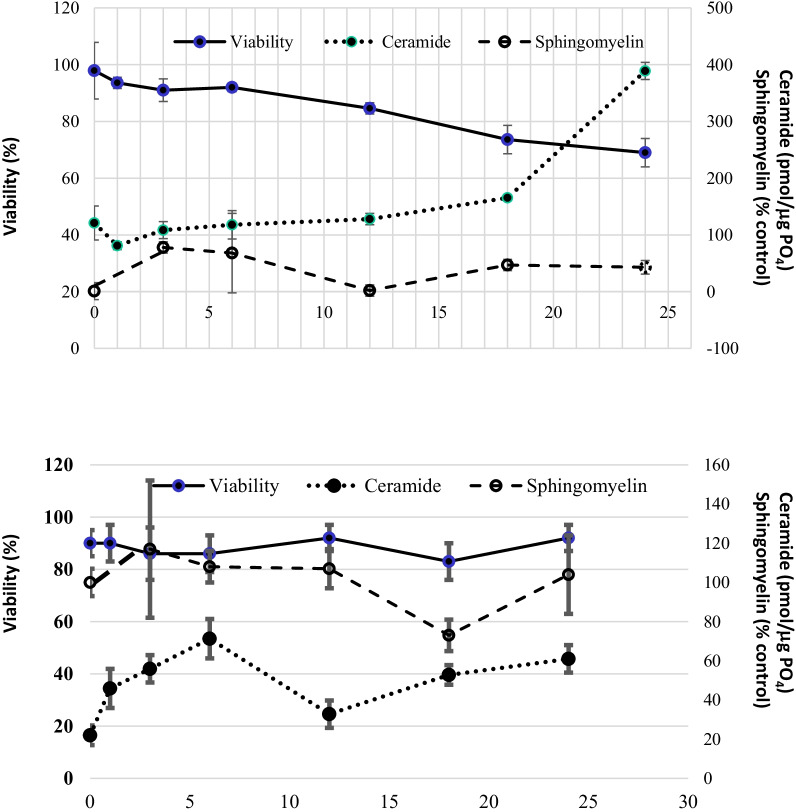
Response to NGF withdrawal in PC12 or TrkA cells. Differentiated PC12 (UPPER) and Trk A (LOWER) cells were deprived of NGF for up to 48 h and collected every 3–6 h for viability assay as well as extraction and quantification of lipids. Quantification of apoptosis (viability) was performed by trypan blue staining of dead cells as well as by staining the cells with the DNA specific fluorochrome Hoechst 33,258 as described in Materials and Methods. A minimum of 250 cells was scored for the incidence of apoptosis in each experiment. Endogenous ceramide was quantified by DAG kinase assay and sphingomyelin by SM mass assay as described in Materials and Methods. Each value represents the mean of duplicate determinations from at least three independent experiments. bars, SEM

When previously-present NGF is removed from the culture medium, approximately 50% of the PC12 cells die (Fig. 5 upper), and sphingomyelin doubles by 12 h, followed by an approximate doubling (at least) of ceramide by 24 h, suggesting that hydrolysis-driven production of ceramide leads to cell death. Ceramide and sphingomyelin levels change little for TrkA cells deprived of NGF, confirming their endogenous stimulation (Fig. 5 lower).

**Fig. 6:**
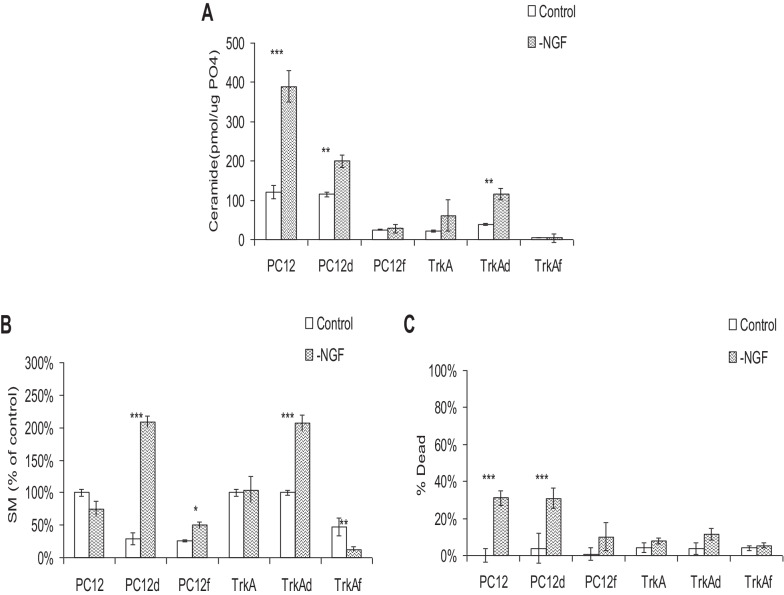
The effect of specific ceramide inhibition on PC12 or TrkA cells following NGF removal. Differentiated PC12 and TrkA cells were deprived of NGF for 24 h in the presence of either 15 μM desipramine or 25 μM FB_1_. Control cells treated with vehicle or inhibitors alone are in white, cells deprived of NGF are depicted with hatching. Each value represents the mean of duplicate determinations from three independent experiments; bars, SEM; *p < 0.05, **p << 0.05, ***p << 0.01. **A** Measurement of ceramide levels. Lipids were extracted and quantified by DAG kinase assay as described in Materials and Methods. **B** Measurement of SM levels. Lipids were extracted and quantified by SM mass assay as described in Materials and Methods. **C** Measurement of viability. A minimum of 250 cells was scored by trypan blue exclusion for the incidence of apoptosis in each experiment

When we withdrew NGF in the presence of inhibitors, we found, as previously, that lack of NGF led to death of PC12 cells but not TrkA cells (Fig. 6). Desipramine failed to protect the cells, but led to a relative doubling of sphingomyelin at 12 h, and a modest increase in ceramide, approximately half of what it would have been without the inhibitor. Fumonisin B_1_, on the other hand, protected the NGF-deprived PC12 cells (death approximately 1/3 that of control), suppressing the rise in both sphingomyelin and ceramide. Overall, these results suggest that the ceramide that can kill cells comes from the hydrolysis of sphingomyelin. As expected, changes in TrkA cells were relatively modest and the cells did not die.

## Discussion

Changes in cell behavior, whether differentiation or apoptosis, can be divided into three phases**:** induction, activation, and acquisition of signature biochemical and morphological features. Ceramide has emerged as a controversial second messenger inducing varied signals leading variously to differentiation, growth, and cell death. Since many distinct pathways can generate ceramide, the origin and perhaps intracellular location of ceramide is likely to determine its downstream signaling activity. Here we investigated what happens to ceramide as rat pheochromocytoma (PC12) and TrkA (Trk 6–24 PC12) cells differentiate or die as controlled by nerve growth factor.

Naive PC12 and TrkA cells respond differently to NGF stimulation. We confirm here that NGF stimulation activates sphingomyelin hydrolysis as well as ceramide synthesis in both cell lines. Small amounts of ceramide are generated by sphingomyelin hydrolysis in TrkA cells, suggesting that TrkA receptor-mediated inhibition of sphingomyelin hydrolysis does not occur in this system; that TrkA-mediated inhibition of sphingomyelin hydrolysis is leaky; or that we have not resolved a potential difference between acid sphingomyelinase, the presumptive target of desipramine, and neutral sphingomyelinase. Although TrkA cells differentiate in response to NGF, they are unable to generate as much ceramide as PC12 cells. PC12 cells exhibit a twofold increase in ceramide prior to initiating differentiation in response to NGF stimulation, and they complete differentiation only after an additional fivefold increase in cellular ceramide. In contrast, naïve TrkA cells have already initiated differentiation even before administration of NGF. Glassman et al. [24] reported that overexpression of TrkA led to receptor dimerization and potentiation of the NGF signal. We report here that TrkA cells complete differentiation with only a twofold increase in cellular ceramide. We conclude that minimal amounts of ceramide are required for progression of differentiation, whereas larger amounts are required for the initiation of differentiation. This is important as high amounts of ceramide activate p38 and JNK/c-Jun actin to induce apoptosis in neurons and other cell types [32, 33].

NGF-induced initiation of differentiation in PC12 cells is regulated by sphingomyelin hydrolysis, but not ceramide synthesis, as desipramine blocks both ceramide elevation and differentiation. This is in agreement with Herget et al. [34] that retinoic acid-induced differentiation is not regulated by ceramide synthesis. However, we report that NGF-induced progression of differentiation in TrkA cells is regulated by both sphingomyelin hydrolysis and ceramide synthesis, since both desipramine and FB_1_ block both ceramide elevation and differentiation (Fig. 1). These results suggest that during differentiation most ceramide is derived from plasma membrane sphingomyelin, with smaller quantities synthesized in cytoplasmic organelles. Furthermore, while both forms of ceramide mediate progression of differentiation, only plasma membrane-derived ceramide initiates NGF-induced differentiation. We suggest that the large increase in sphingomyelin might play a role in neurite outgrowth since sphingomyelin is an important structural component of membranes [35]. During the first two days of differentiation in control PC12 cells, sphingomyelin increases substantially until it is nearly 300–350% of original content. However, as ceramide levels increase sphingomyelin levels drop by a third. Sphingomyelin content in NGF-induced TrkA cells also increases as differentiation progresses, reaching nearly 300–350% of original content by day 4 but there is no comparable drop in sphingomyelin as ceramide levels increase. The increased accumulation of SM in both PC12 and TrkA cells is most likely fueled by ceramide synthesis since the presence of FB_1_ in either cell blocks SM accumulation. Nevertheless, FB_1_ inhibits progression of differentiation only in TrkA cells. Thus**,** sphingomyelin accumulation in itself appears unnecessary for progression of differentiation and furthermore the capacity to generate ceramide in the absence of ceramide synthesis and thus to complete differentiation, is present only in PC12 cells.

In contrast to stimulation of growth and differentiation, apoptosis induced in PC12 cells deprived of NGF was mediated by de novo ceramide synthesis alone and not sphingomyelin hydrolysis, which suggests that these two pathways are specific to downstream events in the PC12 cell line. This finding is in general agreement with a reported finding that ceramide, generated by distinctly different mechanisms, mediates induction of either apoptosis or differentiation by retinoic acid [32]. Nevertheless, TrkA cells respond to NGF withdrawal in the same way they respond to NGF stimulation, namely by activation of both ceramide pathways, which again suggests that these two pathways may substitute for one another [34] although ultimately TrkA cells do not die when NGF is withdrawn.

Although control differentiated TrkA cells have roughly 20% as much ceramide as differentiated PC12 cells, their resistance to apoptosis apparently is not due to an inability to synthesize ceramide. Differentiated TrkA cells can generate ceramide as their ceramide content doubles when NGF is removed in the presence of desipramine. Furthermore, both ceramide pathways appear to be functional in TrkA cells as both are activated by NGF deprivation. Increased ceramide levels are observed during apoptosis following by growth factor withdrawal in PC12 cells [36] and after lethal ischemia in the gerbil hippocampus [37]*.* Furthermore, apoptosis can be induced by exogenous ceramide treatment of PC12 cells [38], mesencephalic [39], hippocampal [40] or cortical neurons [41]. All of this research suggests that apoptosis is dependent on stimulation by an abundance of ceramide.

It is possible that the loss of sphingomyelin that produces the apoptotic phenotype profoundly alters the fluidity of the plasma membrane [42]. However, we have not found that apoptosis is caused by loss of sphingomyelin in either of our cell lines as little sphingomyelin is lost following NGF deprivation. Although sphingomyelin hydrolysis is activated when PC12 cells are deprived of NGF, blocking only ceramide synthesis prevents apoptosis.

In neonatal sympathetic neurons, TrkA and p75^NTR^ function antagonistically for cell survival [16]. TrkA is active in promoting cell survival signals through PI3K/ Akt signaling [4, 5] and suppression of c-Jun phosphorylation and Bax activation [42, 43] while p75^NTR^ contains an intracellular death domain that stimulates both ceramide generation and downstream signaling of JNK [16]. p38, c-Jun phosphorylation by JNK, and MAPK are key regulators of the apoptotic program in post-mitotic neurons after growth factor withdrawal [44]. Since ceramide is generated by sphingomyelin hydrolysis in the plasma membrane and on lysosomal membranes, we speculate that in PC12 cells, ceramide in the plasma membrane signals apoptosis and ceramide in the lysosomal membrane signals differentiation. Ceramide generation via SMase been implicated in cell death caused by anti Fas/CD95, TNFα, IL-1, IFN-γ, vitamin D3, ionizing radiation, heat shock and oxidative stress [35, 36].

## Conclusions

In conclusion, differentiation in PC12 cells and TrkA cells is dependent on sphingomyelin hydrolysis, whereas TrkA cells also rely on ceramide synthesis, and NGF-deprivation induced apoptosis in these cells is also dependent on ceramide synthesis. The interactions of signal molecules with caspases actually define two distinct pathways of apoptosis. One pathway involves the activation of death receptors including Fas, TNF and TRAIL receptors. The second pathway is the mitochondrial pathway. The two ceramide pathways can be distinguished by these two styles of apoptotic mechanisms. In the first, activation of apoptosis by Fas or TNF receptor mediation results in SM hydrolysis and ceramide generation in the plasma membrane. In the second, ceramide synthase activity occurs in the endomembranes, including the mitochondria, by the conversion of sphinganine to dihydroceramide [45, 46].

Although ceramide induced cell death is independent of the Fas ligand/caspase pathway in specific systems [47], in others stress-induced apoptosis mediated by either anti-CD95 (anti-Fas), anti CD40 or TNFα is preceded by increased ceramide generation that is not of SMase origin [48]. Anti-Fas may transiently elevate ceramide followed by downstream sphingosine production, as well as mitochondrial and caspase activation which is then followed by a sustained ceramide accumulation. Caspase inhibitors block both early and later ceramide accumulation in these cells [49] suggesting both that ceramide is not an initiating signal for apoptosis and that ceramide may act as a signal for more than one event during apoptosis.

Our evidence confirms that ceramide is not an initiating signal for apoptosis. Rather, a cell must be primed for apoptosis that is triggered by elevations in cellular ceramide. It is not only an issue of how much of a ceramide signal is present but also in which compartment it is synthesized. Ultimately, we will need techniques that will allow us to localize in real time ceramide in its various compartments.

## Data Availability

All data are available from the corresponding author, ZZ.

## Abbreviations

Akt: AKR mouse strain Thymoma (protein kinase B)
ATP: Adenosine tri-phosphate
aSMase: Acidic sphingomyelinase
D-e-C8-cer: N-octanoyl-D-erythro-sphingosine
DMEM: Dulbecco’s Modified Eagle Medium
FAS: FS-7 (mouse cell line)-Associated Surface antigen
FBS: Fetal bovine serum
G418: Gentamycin
IFN-γ: Interferon gamma
JNK: C-Jun N-terminal kinase
MAPK: Mitogen-Activated Protein Kinase
NGF: Nerve Growth Factor
nSMase: Neutral sphingomyelinase
p75^NTR^: 75 KiloDalton NeuroTrophin Receptor
PC-12: Phaeochromocytoma cells
PI3K: Phosphatidylinositol kinase
PKB: Protein Kinase B
RPMI: Roswell Park Memorial Institute formulation
TNF: Tumor Necrosis Factor
TRAIL: TNF-related apoptosis-inducing ligand
Trk 6–24 = TrkA: Tropomyosin receptor kinase A-overexpressing PC-12 cells
